# Editorial: Patient-Centered Infertility Care: Current Research and Future Perspectives on Psychosocial, Relational, and Communication Aspects

**DOI:** 10.3389/fpsyg.2021.712485

**Published:** 2021-06-25

**Authors:** Lidia Borghi, Julia Menichetti, Elena Vegni

**Affiliations:** ^1^Clinical Psychology, Department of Health Sciences, University of Milan, Milan, Italy; ^2^Faculty of Medicine, Institute of Clinical Medicine, University of Oslo, Oslo, Norway; ^3^Unit of Clinical Psychology, ASST Santi Paolo and Carlo Hospital, Milan, Italy

**Keywords:** assisted reproduction, psychological distress, couple adjustment, doctor—patient relationship, patient—centered care, heterologous fertilization, communication in medicine

## Why Patient-Centered Infertility Care Matters

Infertility affects a large number of couples worldwide. The use of assisted reproductive technology (ART) to address infertility problems has dramatically increased. However, the context of ART poses challenges at different levels to both patients and clinicians. Key challenges for patients include the low treatment success rates, the psychological distress due to the diagnosis of infertility, and the emotional and physical demands of treatments. Not least, under these circumstances, relational bond problems might arise. The ART context also poses challenges to clinicians. Clinicians have frequently to communicate bad news to patients, manage complex interactional consultations with two persons as a patient, address patients' emotions and frequent complaints, and handle couple's treatment discontinuation. Given these complexities, monitoring and improving the quality of fertility care is a priority.

Centering the consultation and care process on the patients' needs and values (i.e., patient-centered care) is one of the key elements for improving quality of care. Good clinician-patient communication and caring relationships are crucial for providing patient-centered care. In the ART field, preliminary research has defined what “patient-centered care” is from the patients' perspective (van Empel et al., 2010) and some studies have identified the main characteristics of clinician-couples' verbal communication during clinical consultations (Leone et al., 2018). However, important knowledge gaps remain in this field, concerning the psychological status of couples undergoing ART treatment, clinician-patient communication, and relational specificities featuring ART care. Such knowledge may help clinicians and healthcare organizations providing patient-centered care to couples affected by infertility problems and receiving ART treatments.

## Ensuring Patient-Centered Infertility Care By Taking Care of The Couples' Psychological Needs

In the scientific literature, it is frequently reported that infertility care can easily bring distress and anxiety to couples (De Berardis et al., 2014). Efforts have been made to explore and better understand the psychological needs of patients who undergo ART treatments (Dancet et al., 2010).

As the contributions in the current Research Topic testify, the psychological well-being, suffering, and adjustment of couples who undergo ART treatments or become parents after a successful ART therapy are crucial areas to explore in order to enable the psychological world of these couples to become visible. In the brief research report in this issue, Zurlo et al. ascertained the protective role of couples' coping strategies in moderating the association between infertility-related stress and anxiety symptoms. In the same issue, Molgora et al. pointed out the importance of considering individual needs as well as enhancing a sense of partnership to improve couples' well-being, taking also into account the gender-related differences that men and women may bring. The importance of taking into account the complexity of the psychological needs of couples has been also underlined by Vasta and Girelli in their perspective paper in the present issue. They propose an approach for addressing couples' needs based on a “matterpsychic” perspective: a model epistemologically close to the biopsychosocial approach suggesting that psychological care should be integrated in a multidisciplinary work.

Two other contributions in this issue rise attention on other crucial but often neglected psychological characteristics of specific groups of ART patients. Di Mattei et al. focus on a specific population of women who might possibly undergo ART treatments: women with cancer who want to access oncofertility preservation. The authors provide indications about particularly resilient psychological characteristics of this group of patients, with functional personality traits and defensive styles. Paterlini et al. performed a longitudinal investigation of parental mental representations during pregnancy and in the post-partum; they revealed that the parental representations of couples who conceived after ART treatments differed and were in general more positive compared to spontaneous conceiving parents.

## Supporting Patient-Centered Infertility Care Through Attention on Clinicians' Challenges

As anticipated, clinicians working in the ART field possibly deal with a variety of psychological, communication and interactional challenges. In previous studies, the attention has been mostly placed on helping clinicians dealing with difficult communication like delivering bad news (Leone et al., 2017). In this issue, Facchin et al. provide an in-depth exploration of difficulties that clinicians experience when caring for couples with infertility problems: from challenges in team working, to difficulties in offering complex therapies that evoke “omnipotence” and that may make errors or failure be often neglected, or in being able to empathically relate with couples.

## Patient-Centered Infertility Care is A Matter of Good Clinician-Patient Communication

Poor communication and relationships with ART clinicians is a cause of dissatisfaction for patients and a reason for discontinuing treatments or changing clinic (Gameiro et al., 2012). Different communication aspects may affect ART care, like insufficient or poor explanations of fertility problems (Gameiro et al., 2012), inadequate information provision and coordination of care (Haagen et al., 2008), or lack of empathy and poor ability to handle psychological distress (Olivius et al., 2004). In this issue, some of these dimensions are explored from new perspectives.

Mosconi et al. performed a literature review on studies tackling the communication of the diagnosis of infertility, as one of the bad news ART doctors have to deliver. They found that this is a quite unexplored topic, with only four studies addressing it in some collateral way. Three articles in this issue analyzed videos of doctor-couple interactions, and highlighted communication specificities in ART visits. Menichetti et al. explored the communication of uncertainty in ART consultations by analyzing the doctors' expression “I don't know”. They revealed how ART doctors may frequently express lack of knowledge, especially about costs and treatment-related aspects, and how patients actually contribute to these expressions by eliciting them and, in some cases, following up. Poli et al. considered another fringe topic in ART dialogues: the presence of laughs and jokes. They found that laughs and jokes are frequently used during ART visits, covering topics related to health status, infertility treatment, organizational aspects, and the doctor-patient relationship. Rossi et al. explored problems of understanding in ART triadic consultations, and concluded that misunderstandings are particularly frequent, especially during the history-taking moments of first visits. Misunderstanding during follow-up consultations, while less frequent, may unveil residual doubts from the couple, especially concerning treatments.

## The Way Forward

The variety of contributions included in this Research Topic testifies the complexity of psychological, interactional, and communication aspects in the care of couples who undergo ART treatments. This highlights the need to systematize such knowledge in evidence-based indications and training for clinicians working in ART care to handle this multiplicity of needs. There has still a lack of studies focused on psychosocial and communication challenges involved in the heterologous fertilization and gamete donation for oocyte recipients. The few studies on the topic reported inconsistent results regarding the emotional distress experienced by those women (Bracewell-Milnes et al., 2016). Future studies may be needed to explore the emotional experience of couples who specifically undergo heterologous fertilization. Similarly, clinicians' challenges and clinician-patient communication in the field of heterologous fertilization should be addressed.

## Author Contributions

LB, JM, and EV conceived the idea, manage the topic, and wrote the manuscript. All authors contributed to the article and approved the final version.

## Conflict of Interest

The authors declare that the research was conducted in the absence of any commercial or financial relationships that could be construed as a potential conflict of interest.
